# Physicochemical Methods for the Structuring and Assembly of MOF Crystals

**DOI:** 10.1021/acs.accounts.4c00250

**Published:** 2024-07-26

**Authors:** Tolga Zorlu, Daniel Hetey, Michael R. Reithofer, Jia Min Chin

**Affiliations:** † Department of Functional Materials and Catalysis, University of Vienna, Währinger Straße 42, 1090 Vienna, Austria; ‡ Department of Inorganic Chemistry, University of Vienna, Währinger Straße 42, 1090 Vienna, Austria

## Abstract

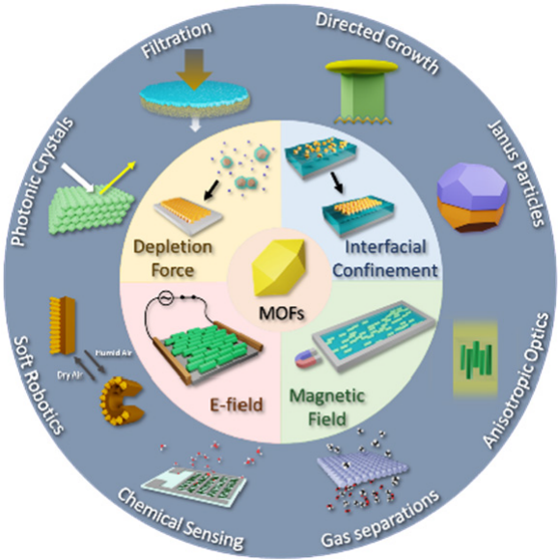

Metal–organic frameworks (MOFs) are promising for various applications through the creation of innovative materials and assemblies. This potential stems from their modular nature, as diverse metal ions and organic linkers can be combined to produce MOFs with unique chemical properties and lattice structures. Following extensive research on the design and postsynthetic chemical modification of MOF lattices at the molecular level, increasing attention is now focused on the next hierarchical level: controlling the morphology of MOF crystals and their subsequent assembly and positioning to create functional composites.

Beyond well-established methods to regulate crystal size and shape through nucleation and coordination modulation, physicochemical techniques leveraging wetting effects, interparticle interactions, and magnetic or electric fields offer attractive avenues for the hierarchical structuring and assembly of MOFs. These techniques facilitate crystal alignment and yield unique superstructures. While our research group primarily focuses on directing MOF crystal orientation and positioning using external stimuli such as magnetic and electric fields, we also explore hierarchical MOF synthesis and structuring using liquid interfaces and depletion force-assisted packing.

This account highlights our journey and progress in developing methods to regulate the morphology, assembly, orientation, and positioning of MOF crystals, placed in the context of work by other groups. First, we examine commonly utilized structuring methods for MOF crystals that employ liquid–liquid and air–liquid interfaces to spatially confine reactions, allowing us to access unique morphologies such as mushroom-like crystals and Janus particles. We also discuss strategies for concentrating and packing MOF crystals into superstructures, utilizing fluid interfaces for spatial confinement of crystals, depletion forces, entropic effects, and crystal sedimentation.

A particularly compelling challenge in expanding the applicability of MOF materials is how to manipulate free-standing MOF crystals. This issue is especially important because MOFs are typically produced as loose powders, and industrial material processing is generally more efficient when the material is fluidized. While extensive research has been conducted regarding MOF growth on substrates with both positional and orientational control, there is a clear need for similar precision with free-standing MOFs dispersed in a fluid matrix. Our group has thus focused on the relatively new, yet powerful approach of using electric and magnetic fields to manipulate MOF crystals, which offers unprecedented control over the orientation and positioning of dispersed MOF crystals, complementing the more well-established methods of MOF growth on substrates. In this Account, we provide foundational background and discussions on the interactions between these external fields and MOF crystals, including critical considerations for effective MOF manipulation using such techniques. We also discuss their unique advantages and applications, and briefly examine potential application areas, such as photonics, smart materials like soft robotics and absorbents, and sensing. This Account highlights the promising potential of well-organized and aligned MOF crystals over randomly oriented ones in various applications, owing to enhanced selectivity and performance. It underscores the importance of specialized assembly methods to advance materials science and engineering, encouraging the reader to explore such approaches.

## Key References

- Allahyarli, K.; Reithofer, M. R.; Cheng, F.; Young, A. J.; Kiss, E.; Tan, T. T. Y.; Prado-Roller, A.; Chin, J. M. Metal–Organic Framework superstructures with long-ranged orientational order via E-field assisted liquid crystal assembly. *J. Colloid Interface Sci.***2022**, 610, 1027–1034.^1^ This work shows the assembly of MOF crystals into nematic superstructures through electrical-field assisted sedimentation.
- Cheng, F.; Young, A. J.; Bouillard, J.-S. G.; Kemp, N. T.; Guillet-Nicolas, R.; Hall, C. H.; Roberts, D.; Jaafar, A. H.; Adawi, A. M.; Kleitz, F.; Imhof, A.; Reithofer, M. R.; Chin, J. M. J. Dynamic electric field alignment of metal–organic framework microrods. *J. Am. Chem. Soc*. **2019**, 141 (33), 12989–12993.^2^ This study demonstrates that solvent-suspended NU-1000 microrod crystals can align very rapidly upon exposure to electric fields and thus have the potential to be integrated into a variety of electronic and optical systems.
- Cheng, F.; Marshall, E. S.; Young, A. J.; Robinson, P. J.; Bouillard, J. S. G.; Adawi, A. M.; Vermeulen N. A.; Farha, O. K.; Reithofer, M. R.; Chin, J. M. Magnetic control of MOF crystal orientation and alignment. *Chem. Eur. J*. **2017**, 23 (62), 15578–15582.^3^ This study sheds light on the magnetic orientation of MOFs such as NH_2_-MIL-53(Al) and NU-1000 whose surfaces are enriched with magnetic nanoparticles.
- Tan, T. T.; Reithofer, M. R.; Chen, E. Y.; Menon, A. G.; Hor, T. A.; Xu, J.; Chin, J. M. Tuning Omniphobicity via Morphological Control of Metal–Organic Framework Functionalized Surfaces. *J. Am. Chem. Soc*. **2013**, 135 (44), 16272–16275.^4^ This work demonstrates the use of the liquid–air interface to localize epitaxial MOF growth and generate unique MOF micromushroom structures which impart omniphobicity to the surface.

## 1 Introduction

Extensive research into metal–organic frameworks (MOFs) has led to a diverse array of such materials, boasting distinct pore sizes, morphologies, and porosities that find utility across a broad spectrum of disciplines such as sensing,^5^ catalysis,^6^ and separations.^7,8^ Following on from the chemistry of MOFs at the molecular level, there is a growing interest in advancing to the next hierarchical stage of MOF control: managing the morphology and assembly of MOF crystals. Specifically, utilizing physicochemical methods that harness wetting effects, interparticle interactions, and magnetic and electric fields offers promising pathways for structuring and assembling MOFs hierarchically.

In this Account, we highlight some examples from our group and that of others regarding the use of fluid interfaces for the generation of unique MOF structures such as Janus particles and “mushroom-like” structures, as well as the formation of MOF crystal assemblies via packing at fluid interfaces, through entropic interactions as well as with external field assistance, simplifying the key concepts behind such methods for the general reader and examine some potential applications for which the resulting MOF materials can be employed.

## 2 General Structuring and Assembly Methods for MOF Crystals

Control over MOF crystals and their assembly into ordered superstructures requires physical forces in one form or another to direct their crystal orientation, packing, and positioning. In general, the current approaches for the self-assembly of MOF crystals can classified into several major categories, namely, (i) the use of confinement such as within droplets or to fluid interfaces to direct the formation of crystal assemblies;^9^ (ii) entropy-directed or depletion force-assisted methods;^10^ and (iii) external-field directed assembly utilizing electric-^1,11^ or magnetic-fields.^12−14^ We highlight a few examples from each category below, focusing especially on the use of liquid interfaces for MOF structuring, as well as external-field assisted assembly, which are of particular interest to our group.

### 2.1 Fluid Interface-Assisted Structuring and Assembly

**Figure 1:**
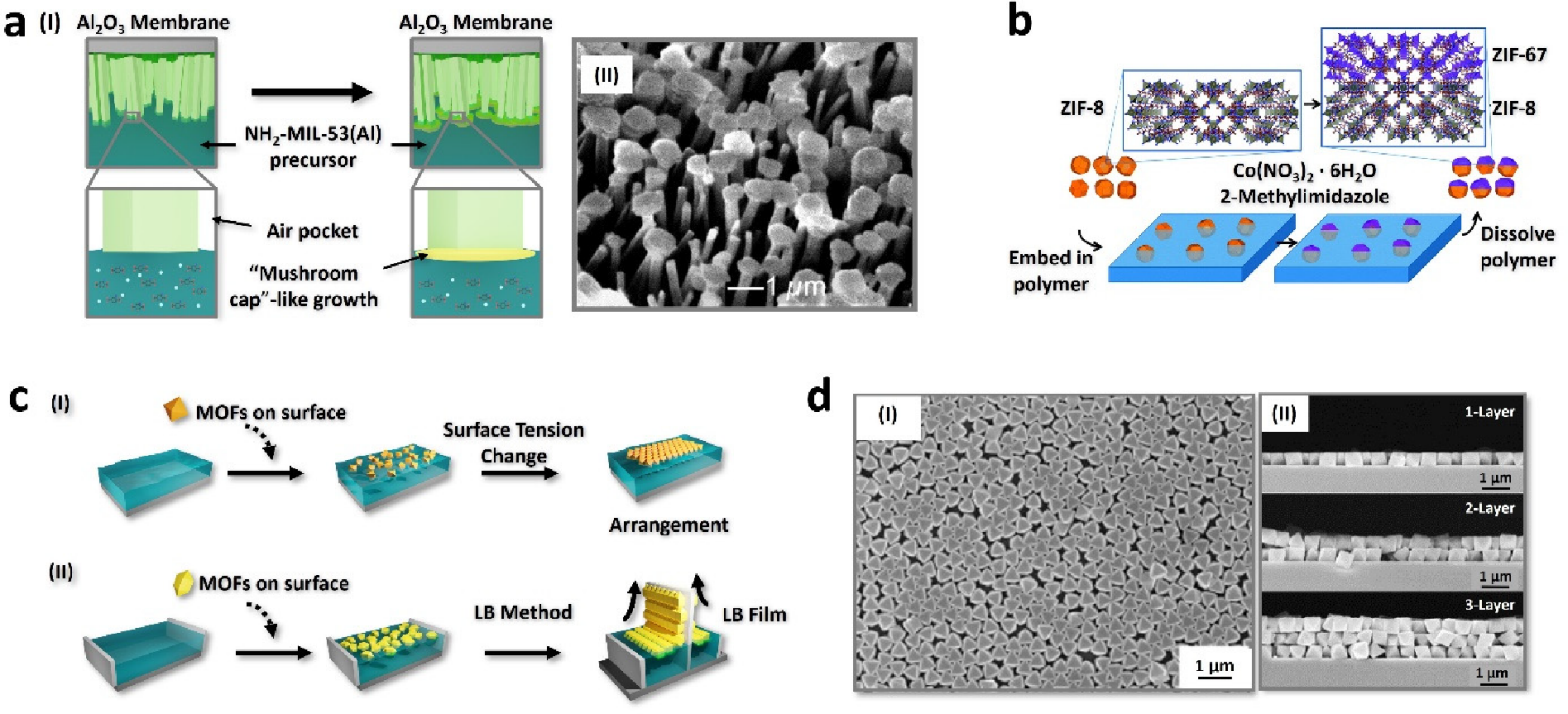
a) Schematic illustration (I) and SEM image (II) of mushroom-like NH_2_-MIL-53(Al) MOF synthesis through interfacial epitaxial growth. Adapted with permission from ref (4). Copyright 2013, American Chemical Society. b) Schematic illustration of Janus MOF crystals preparation, where ZIF-8 crystals are partially embedded in PMMA (blue). Subsequent modification of the exposed portion and polymer removal affords Janus particles. Reprinted with permission from ref (18). Copyright 2014, Royal Society of Chemistry. c) Schematic illustration of the fluid interface-assisted assembly approach via surface tension changes in the solvent (I), and MOF superstructures obtained through the well-known LB method (II). d) Scanning-electron microscopy (SEM) image of the UiO-66 crystal superstructure on a silicon substrate (I), and the cross-sectional SEM image taken after 1–3 repetitions of the procedure (II). Adapted with permission from ref (22). Copyright 2013, John Wiley and Sons.

Air–liquid and liquid–liquid interfaces have been widely utilized for the preparation of MOF materials with unique structures, as confinement of reactions and materials to the interface offers control over crystallizations, deposition, and growth.^15^ Our group exploited the use of the water–air interface to generate MOF mushroom-like structures, whereby the special mushroom-like morphology of the MOF crystals afforded the crystals “*re-entrant topography*”^16^ which rendered them with unique oleophobicity^4^ (Figure 1a). We initially prepared densely grown NH_2_-MIL-53(Al) microneedles on an anodic aluminum oxide membrane substrate, whereby evolutionary selection led to their ⟨001⟩ preferred orientation perpendicular to the substrate. Chemical functionalization of the microneedle arrays with perfluorooctanoyl chloride rendered the samples superhydrophobic, but not oleophobic, as demonstrated by their complete wetting by hexadecane. To obtain oleophobicity, it was necessary to general special “re-entrant textures” by manipulating the surface morphology. The microneedle arrays were inverted and placed on the surface of an aqueous MOF precursor solution, whereby the superhydrophobicity of the material limited liquid wetting and therefore subsequent epitaxial growth of NH_2_-MIL-53(Al) “*caps*” at the microneedle tips. This resulted in the formation of mushroom-like structures that demonstrated oleophobic behavior with nonpolar solvents such as diiodomethane and hexadecane after further fluorination while further growth led to fusion of the “*caps*” into a NH_2_-MIL-53(Al) film on the microneedles. Besides air–liquid interfaces, liquid–liquid interfaces allow the confinement of MOF growth and the selective positioning of preformed MOF crystals at the interface. Confinement of particles at an oil–water interface and subsequent solidification of one of the liquid phases to partially mask the particles is a common technique to generate Janus colloids.^17^ In the first example of Janus MOF micromotors,^18^ we suspended ZIF-8 particles at the interface between a solution of poly(methyl methacrylate) (PMMA) in ethyl acetate and water (Figure 1b). Upon evaporation of the ethyl acetate, PMMA films bearing partially exposed ZIF-8 crystals were obtained. Subsequent heteroepitaxial growth of isoreticular ZIF-67 onto the exposed faces of the ZIF-8 crystals in the presence of poly(vinylpyrrolidone) (PVP) modulator and the removal of PMMA via dissolution afforded Janus ZIF-8/ZIF-67 crystals. As the Co^2+^-bearing ZIF-67 is redox-active, but the Zn-based ZIF-8 is redox inactive, placement of the ZIF-8/ZIF-67 particles in an H_2_O_2_ solution led to the catalytic decomposition of H_2_O_2_ on the ZIF-67 side, and the bubble-ejection based propulsion of the microparticles through the suspending liquid.

Besides the structuring of MOF crystals, fluid interfaces (air–liquid and liquid–liquid interfaces) offer one of the most well-known and straightforward methods for achieving MOF assemblies.^19−21^ By exploiting the surface energies of the crystals relative to the fluids, crystals can be reliably confined to the interface, restricting their freedom of motion. In the case of highly uniform crystals, their specific arrangements can be achieved through entropy-driven ordered packing. In brief, changes in the interfacial tension of the solvent(s) are utilized to encourage crystals present in a specific solvent to come together near the interface in an organized manner (Figure 1c). In a well-known study, Lu et al. (2013) synthesized UiO-66 crystals with varying sizes and utilized interfacial tension to align these crystals in a two-dimensional arrangement.^22^ To achieve this, they initially coated UiO-66 crystals with PVP, thereby enhancing the stability of the crystals within the dispersant and preventing their aggregation. Following this, the addition of sodium dodecyl sulfate (SDS) to the dispersant altered the surface tension of the liquid phase, facilitating the ⟨111⟩-oriented arrangement of crystals into monolayers (Figure 1d). Moreover, when the monolayers were transferred to a solid platform, and the process repeated multiple times, substrate-supported UiO-66 layers of varying thicknesses could also be obtained.

Another common approach used to obtain MOF superstructures at the liquid interface is the Langmuir–Blodgett (LB) method, which does not require changes in the surface tension of the solvent.^23^ Here, MOF crystals placed on the liquid surface are forcibly brought together using movable lateral barriers and transferred onto a solid substrate (Figure 1c), enabling precise control over the MOF film thickness and the crystal arrangement.

### 2.2 Controlled Drying and Depletion Force-Assisted Assembly

**Figure 2:**
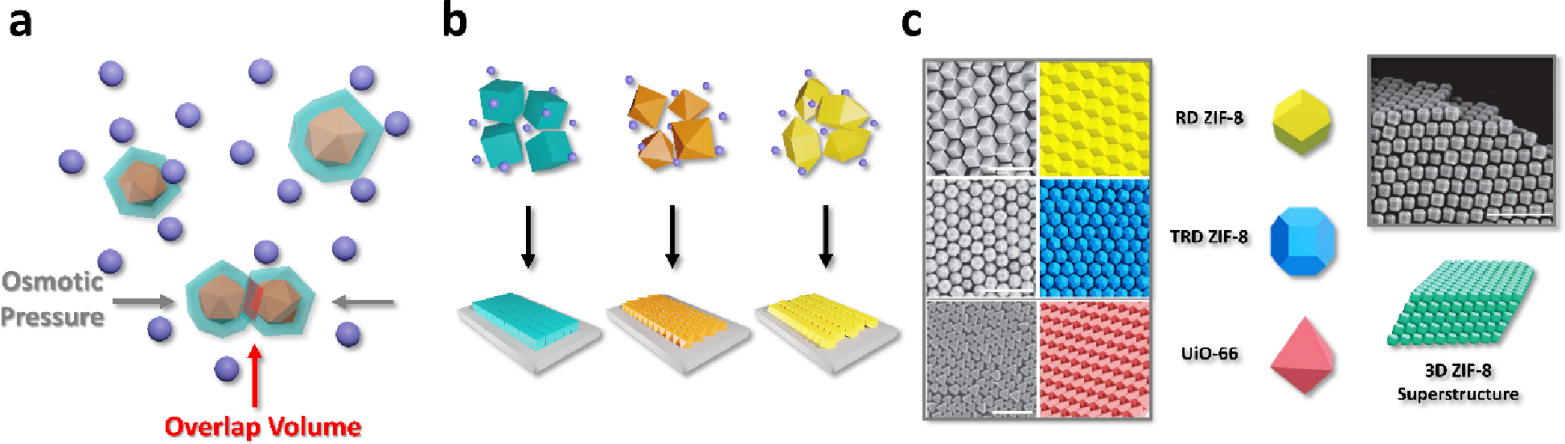
a) Schematic illustration of depletion force. b) Schematic illustration of the preparation of MOF assemblies through the utilization of the depletion force-assisted strategy. c) MOF superstructures based on different particle morphologies. Scale bars: 1 μm. Adapted with permission from ref (26). Copyright 2018, Nature Springer.

An alternative to spatial confinement at fluid interfaces is the use of controlled drying processes, which may also be accompanied by the utilization of depletants (Figure 2a). Depletants refer to noninteracting solutes such as nonadsorbing polymers (represented by purple spheres) which are physically excluded from the immediate vicinity of larger colloidal particles such as MOFs (represented by orange polyhedrons). If such solutes are approximated as small spheres, the centers of the spheres cannot approach the MOF crystals closer than the exclusion zone, depicted as a turquoise shell area around the orange MOF polyhedral cores. The drive to minimize the system free energy and increase the remaining free volume for depletant occupation favors the overlapping of depletion layers, observed as a directional osmotic pressure exerted by the depletants upon the MOF crystals. (Figure 2a) This results in an apparent attraction between the MOF crystals, encouraging their assembly into diverse superstructures.^19,24,25^

Based on their morphology, MOF crystals can be aligned in specific directions, or organized into 3D or 2D arrays upon drying. For instance, Avcı et al. (2018) systematically synthesized submicrometer-size UiO-66 and truncated rhombic dodecahedral ZIF-8 crystals and subsequently generated oriented 3D assemblies of these MOFs, facilitated by the presence of cetyltrimethylammonium bromide (CTAB) (Figure 2c).^26^ The three-dimensional MOF superstructures were achieved by drying aqueous colloid MOF solutions of different sizes and types of crystals on glass slides at varying temperatures sufficiently low to allow gradual solvent evaporation, leading to a more uniform and homogeneous ordering of the superstructures. The authors subsequently demonstrated the photonic properties of these assemblies, which showed angle-dependent coloration as the lattice periodicity is comparable to the wavelengths of visible light. Although the authors do not explicitly refer to the use of depletion forces, the necessary presence of CTAB for ordered assembly suggests that depletion interactions also play a role. Further, in a recent study, Wang and co-workers (2022) assembled MOF microcrystals with diverse morphologies and compositions, such as ZIF-8, UiO-66, MIL-88A, and MIL-96 into both 3D and also 2D assemblies by employing cetyltrimethylammonium chloride (CTAC) and sodium dodecyl sulfate (SDS) as depletants.^27^ The selective formation of low-dimensional versus 3D assemblies is determined by particle morphology and preferential particle attachment to a smooth substrate which limits the possibilities for facet-to-facet particle attachment, favoring the formation of 1D particle chains which can attach to each other lengthwise to generate staggered 2D assemblies.

Overall, the depletion force-assisted assembly enables highly regular MOF crystal packing on substrates but is constrained by the need for a precise selection of depletant molecules together with meticulous adjustment of their concentrations, as well as the requirement of high MOF crystal uniformity.

### 2.3 External Field-Assisted Assembly

**Figure 3:**
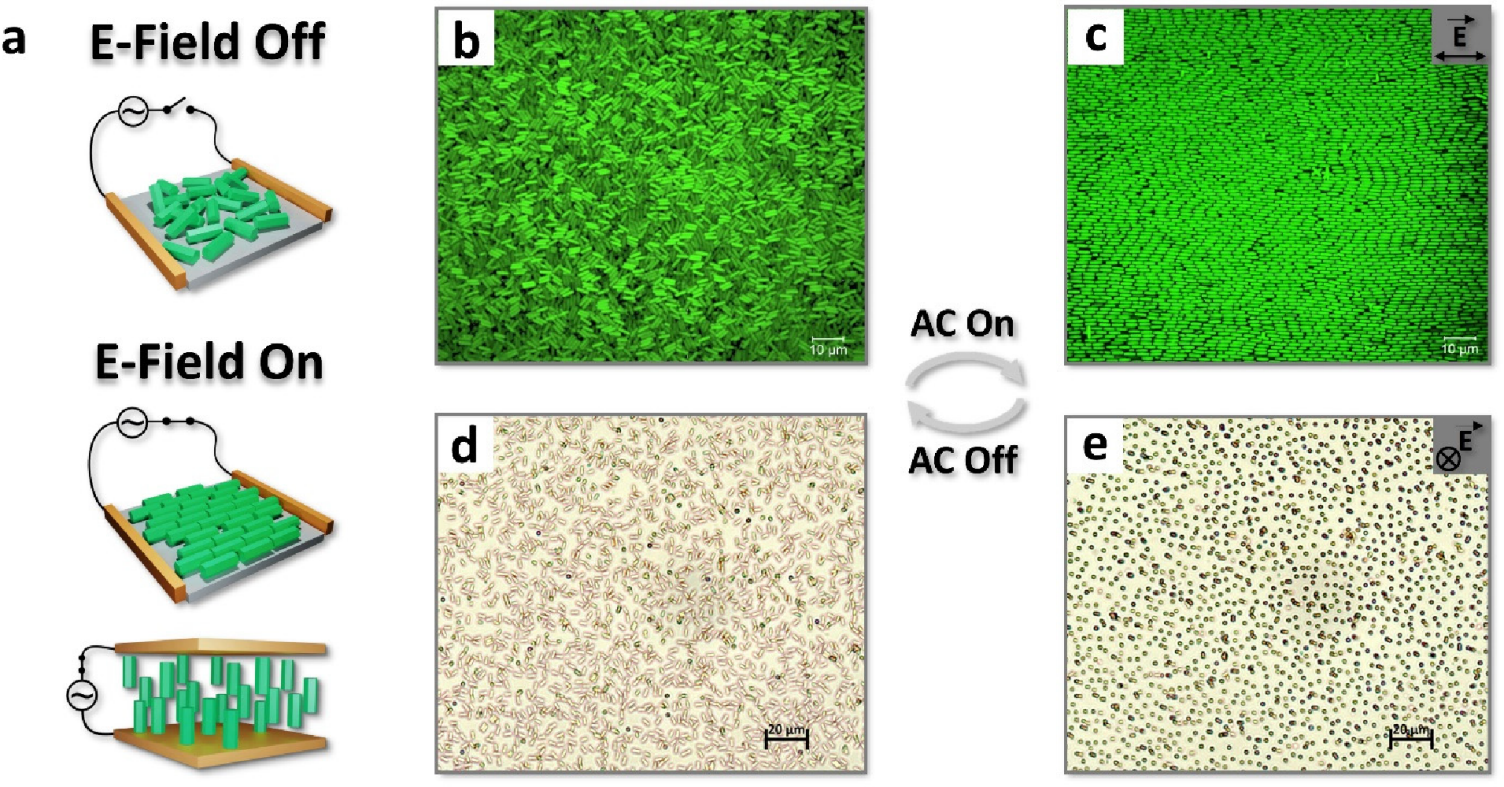
a) Schematic illustration of alignment of anisotropic MOF crystals on a conductive glass substrate under an electric field. b) confocal laser scanning microscopy (CLSM) image of NU-1000 MOF crystals without E-field, and c) with E-field. Adapted with permission from ref (1) under a Creative Commons CC-BY license. d) The crystals with anisotropic morphology, such as NU-1000, can easily and rapidly alter their alignment within a solution in the presence and absence of an E-field.

Assembly techniques leveraging external stimuli, notably magnetic and electric fields, offer compelling avenues for the hierarchical assembly of MOFs. Our research group primarily focuses on directing the orientation and positioning of MOF crystals utilizing these external influences (Figure 3), as these are robust, easily reproducible, and generally applicable methods for manipulating MOF and other colloidal materials.

#### 2.3.1 Electric Field-Assisted Assembly

In an early example, Granick et al. (2013) synthesized monodispersed rhombic dodecahedral particles of ZIF-8, and dispersed the crystals in ethylene glycol.^28^ Confocal microscopy of the dye-functionalized ZIF-8 demonstrated that applying an external E-field to ZIF-8 microcrystals led to ZIF-8 particle chaining into 1-D superstructures. The formation of particle chains in an E-field arises as the E-field induces dipoles in the particles. The polarized particles then interact via dipole–dipole attraction, facilitating chain formation when the dipolar attractions are strong enough to overcome interparticle electrostatic repulsions and randomizing flows due to E-field induced ion migration or heating effects. The dipolar attraction between adjacent particles can be described as follows:
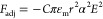
2.1

Here, *C* is a coefficient with a dependence on the distance between particles, *E* is the strength of the E-field, *ε*_m_ refers to the permittivity of the medium, *r* refers to the radius of the crystal (which is approximated as a sphere), and α refers to effective polarizability of the particle, represented by the real part of the Clausius–Mossotti function.^29−31^ It follows that particle chaining can be easily tuned by controlling the strength of the applied E-field, which in turn can be managed by changing the interelectrode distances or the magnitude of the applied potential. In the above-mentioned example, besides dipolar interactions arising due to the external field, van der Waals forces between the flat particle facets of adjacent particles induced particle rotation to maximize interparticle facet-to-facet contact, leading to ⟨110⟩ orientation of rhombic dodecahedral ZIF-8 and ⟨100⟩ orientation of truncated cubic ZIF-8 along the E-field. Like for liquid-interface and depletant-assisted assembly, selective crystal orientation necessitates the use of monodispersed and highly crystalline particles with flat facets of sufficient areas, limiting the applicability of this method as the preparation of highly uniform MOF crystals is difficult to achieve across a wide spectrum of MOFs.

Alternatively, external fields can affect orientational control of anisotropically shaped crystals such as rod-shaped MOF crystals where the ratio of their length versus width, or aspect ratio (AR), is higher than 1. Orientational control over these crystals occurs through the development of dipoles along specific crystallographic axes, whereby the strongest induced dipoles within the crystals align with the applied external field direction as a consequence of minimizing the potential energy. To ensure selective crystal orientation, overcome the rotational inertia arising from the solvent viscosity, and resist thermal randomization, the energy of dipole alignment in the crystals must be sufficient to overcome these opposing forces. Consequently, the energy differentiation induced by external fields between the desired field-aligned and the unaligned states of the crystals can be leveraged to achieve orientational control of MOF crystals.^2^

**Figure 4:**
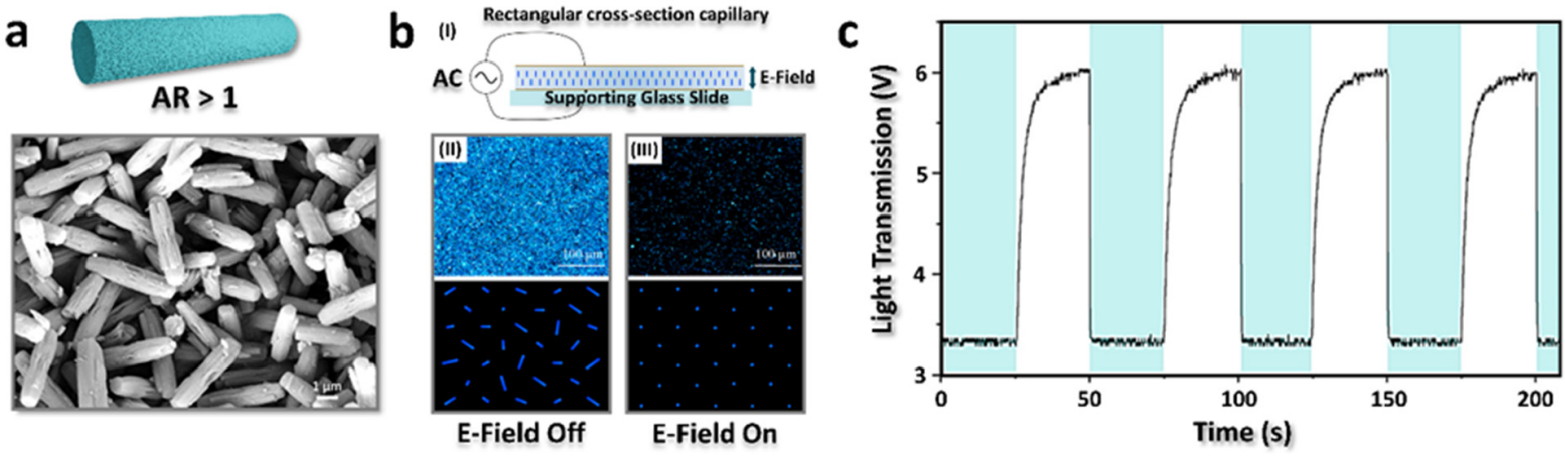
a) Illustration and SEM images of anisotropic MOFs, NU-1000. b) (I) Cross-sectional view of rectangular capillary used in electro-optical measurements; (II) top and bottom, respectively: bright polarized optical microscopy (POM) image and illustration of NU-1000 suspension showing light transmission when E-field is off; (III) top and bottom, respectively: dark POM image and illustration of the same suspension showing decreased light transmission due to NU-1000 E-field alignment. (c) Electrooptical response of the suspension-light transmission under alternating E-field (White: light transmission, cyan: E-field on). Adapted from ref (2). Copyright 2019, American Chemical Society.

For instance, in the first example of orientation via E-field-induced dipoles in MOFs, we demonstrated that MOF crystals with anisotropic morphology, such as trimethoxy(octadecyl)silane (OTS)-functionalized NU-1000 (NU-1000_Si_), rapidly align along an alternating external E-field.^2^ The silanization of NU-1000 was carried out to enhance MOF dispersibility in nonpolar solvents to allow their colloidal manipulation. An advantage of NU-1000_Si_ arises from its intrinsic fluorescence, endowed by its pyrene-derived ligands, allowing their visualization via confocal microscopy without the need for dye-functionalization. NU-1000_Si_ microrod crystals, suspended in bromobenzene, showed strong interparticle electrostatic repulsion owing to the poor screening capability of the nonpolar solvent. Therefore, applying an external alternating electric field led to rapid alignment of the MOF particles along the E-field, but not to particle chaining. The uniaxially birefringent NU-1000 crystals allow light transmission through crossed polarizers on an optical microscope, whereby particle orientation changes their overall light transmission, which was measured via a photodetector to follow the electroresponse of the particle. When the E-field was turned on, particle alignment along the light transmission pathway led to rapid minimization of detected light. Worth noting is that this response was reversible and showed no diminishing after repeated on–off cycles. This capability suggests their potential utility in various sensor or electro-optical applications (Figure 4).

The mechanism behind the crystal alignment relies on the interactions between the E-field, the ions in the system, and the induced dipole moments in the dielectric particles. An E-field generated by alternating current can induce an alternating dipole moment in the particles via microscale charge separation, depending on the effective polarizability, α of the particles, as approximated by the real part of the Clausius–Mosotti equation, represented below:
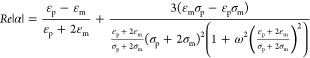
2.2where ε is the relative permittivity, σ is the conductivity of the particles (*p*) or the medium (*m*) respectively and ω is the angular frequency of the applied field. When the induced dipole is large enough to inflict sufficient torque on the particle to overcome viscous inertia and thermal randomization (given by *k*_B_*T*, where *k*_B_ is the Boltzmann constant and *T* the temperature), the particle rotates to align along the field direction.

The induced dipole alignment energy is given by^32^
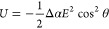
2.3where Δα is the difference in electric polarizability of the particle along and perpendicular to the particle’s main axis and θ is the angle of the rod to the E-field direction. Control over the magnitude of the E-field is an effective method to encourage particle alignment, as seen by the second-order dependence of the alignment energy on it. The polarization of the crystals is also significantly influenced by the alternating current (AC) frequency of the applied field, as seen from eq 2.2.^29^ At low AC voltages, the crystal orientation is primarily dictated by field-induced ion migrations within and around the MOF crystals. These oscillations generate sufficient force to overcome the rotational inertia of the suspended crystals. However, at higher frequencies, the ionic migration can no longer keep up with the field frequency, leading to a shift in the mechanism from ionic flows to other dielectric polarization of the particles.

**Figure 5:**
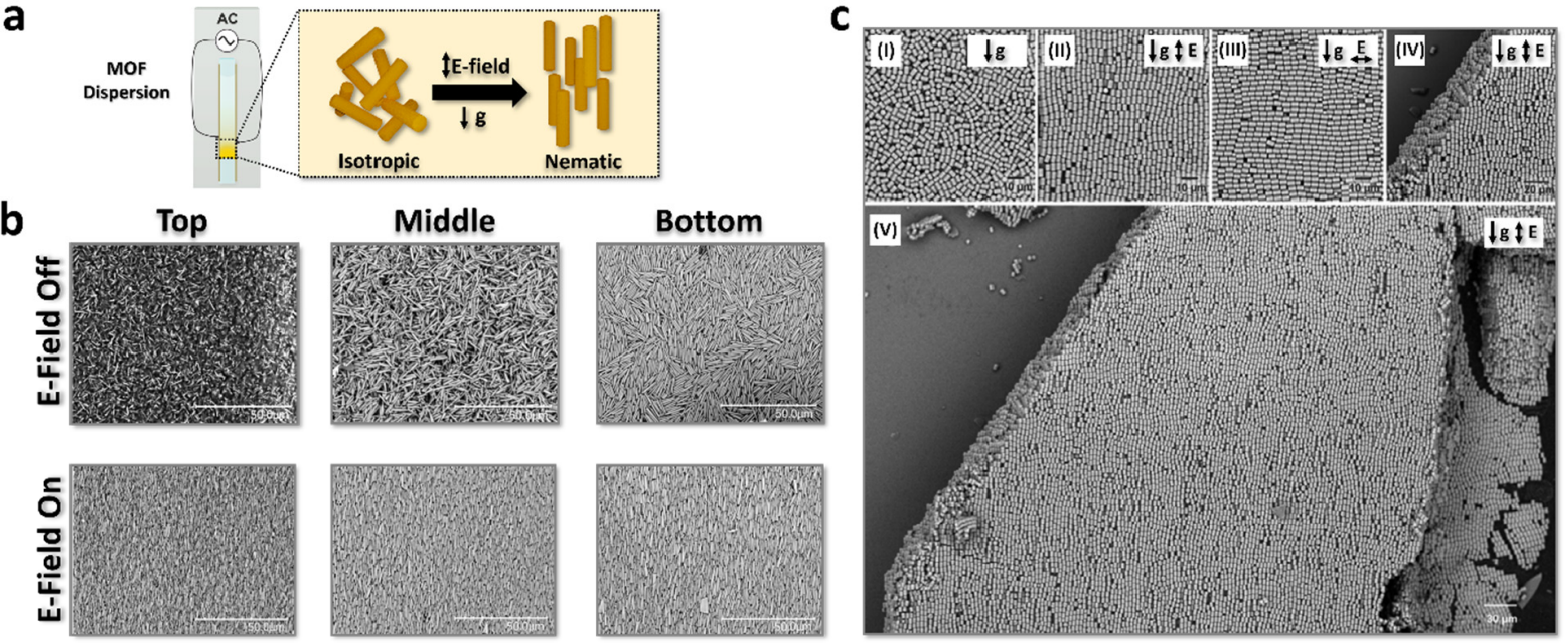
a) Schematic illustration of isotropic and nematic phases of MIL-68(In) dispersion (AR = 6.8) in capillary before and after E-field-assisted sedimentation. b) Corresponding SEM images of MIL-68(In) assemblies at the top, middle, and bottom of the sediment in the absence and presence of an E-field. c) SEM images of opened capillary samples containing short MIL-68(In) (AR = 1.2) sedimented (I) without an E-field; (II) in a vertical E-field; (III) in a horizontal E-field; (IV) in a vertical E-field showing the alignment of lower particle layers; (V) in a vertical E-field showing the long-ranged order of the particles. Adapted with permission from ref (1) under a Creative Commons CC-BY license.

As mentioned earlier, the formation of highly ordered MOF crystal superstructures relies on packing interactions, requiring high crystal size and shape uniformity, thereby presenting a significant synthetic hurdle for scientists. A notable effect observed in rod-shaped, anisotropic colloids with high AR is their ability to pack into liquid crystalline nematic phases with directional order, as proposed by Onsager,^33^ even when polydispersed. Like depletant-assisted assembly, this effect is driven by free volume and entropy maximization. For the transition from the isotropic, randomly oriented phase to the nematic, directionally ordered phase, the volume fraction of the particles must be considered—at low particle volume fractions, the orientational entropy of the particles favors the disordered phase whereas at high volume fractions, free volume entropy favors nematic phase formation. Consequently, the isotropic–nematic phase transition occurs when the particle volume fraction changes.^34^ Typically, sedimentation is utilized to increase the volume fraction of the particles in the suspension to encourage their packing. Careful control over the sedimentation speed is typically necessary to avoid the gelation of disordered phases, with some examples allowed to slowly sediment over the course of a year to ensure ordering.^35^ Another consideration is the particle aspect ratio—theoretical calculations by Bolhuis and Frenkel suggest that rod-shaped particles of aspect ratios exceeding 4.1 and 4.7 are required for nematic and smectic ordering—a requirement unmet by most reported MOF crystals.^36^ Nevertheless, in a study carried out by our group,^1^ rod-shaped MOF crystals of NH_2_-MIL-53(Al), MIL-68(In) and NU-1000 with ARs ranging from 10 to 1.2 were prepared. It was demonstrated by applying an E-field during the sedimentation process, even MOF crystals with a low AR of approximately 1.2 showed nematic packing (Figure 5), demonstrating the wide applicability of this method. Notably, polydispersed NH_2_-MIL-53(Al) crystals could also form oriented assemblies, as directional ordering is not reliant upon particle packing interactions which can be easily disrupted by size and shape mismatching of particles. The E-field assisted approach also allows avoidance of the need for slow, controlled sedimentation as oriented millimeter-scale assemblies can be formed in 20 min (Figure 5c).

Manipulating MOF crystals under the influence of an E-field, and the hierarchical structures arising therefrom, offer advantages in terms of ease of approach, speed, and dynamism and can serve as a postsynthetic method to rapidly and reversibly orient MOF crystals. This technique is robust enough that minor perturbations do not significantly affect crystal alignment, enhancing reproducibility. Furthermore, it can be efficiently downscaled by employing microfabricated electrodes, as seen in the case of interdigitated electrodes used for sensing applications.^37^

#### 2.3.2 Magnetic Field-Assisted Assembly

The manipulation of particles under magnetic influence and the subsequent efforts to achieve controlled orientation and assembly represent an alternative to E-field-assisted assembly.^38^ However, only a small fraction of MOFs possess intrinsic magnetic properties. To overcome this, a simple approach to generating interaction between a nonmagnetic MOF crystal and a magnetic field is by attaching superparamagnetic or ferromagnetic particles via electrostatic attraction to the MOF crystals.^3^ By varying the pH of the particle suspension, the zeta potentials of the particles and hence the interparticle electrostatic attractions can be systematically tuned. We first prepared poly(acrylate)-stabilized magnetic Fe_3_O_4_ nanoparticles which bear a negative zeta potential above pH 2.8, and NH_2_-MIL-53(Al) as well as NU-1000 as representative MOF systems. Below pH 10.5, protonation of the amino groups of NH_2_-MIL-53(Al) endows the crystals with positive charges. By dispersing NH_2_-MIL-53(Al) with the Fe_3_O_4_ nanoparticles in a buffered solution of pH 3.5, the negatively charged Fe_3_O_4_ rapidly attached to the oppositely charged NH_2_-MIL-53(Al), effectively generating a magnetic field responsive shell around the MOF crystals.

The adsorption of superparamagnetic particles onto the external facets of MOF crystals affords them a much stronger magnetic behavior, whereby the exposure of such microrods to a magnetic field exerts a torque upon the particles to minimize their magnetic energy. Similar to E-field alignment, magnetic alignment of the MOF crystals requires that the difference in magnetic energy of aligned and unaligned particles is larger than thermal randomization energy. For rods with AR of >10, the magnetic energy can be approximated by
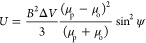
2.5Where *U* is the magnetization energy, *B* is the strength of the magnetic field, Δ*V* is the volume of the magnetic envelope, *μ*_p_ and *μ*_o_ are the magnetic permeabilities of the rod particle and that of free space, and Ψ is the angle between the applied magnetic field and the major axis of the rod.^39^ It can be seen that the magnetic energy is minimized when the rods are aligned along the magnetic field (Ψ = 0) and the energy shows a second-order dependence on the strength of the applied field and a first-order dependence on the volume of the magnetic envelope arising from the adsorbed magnetic particles.

**Figure 6:**
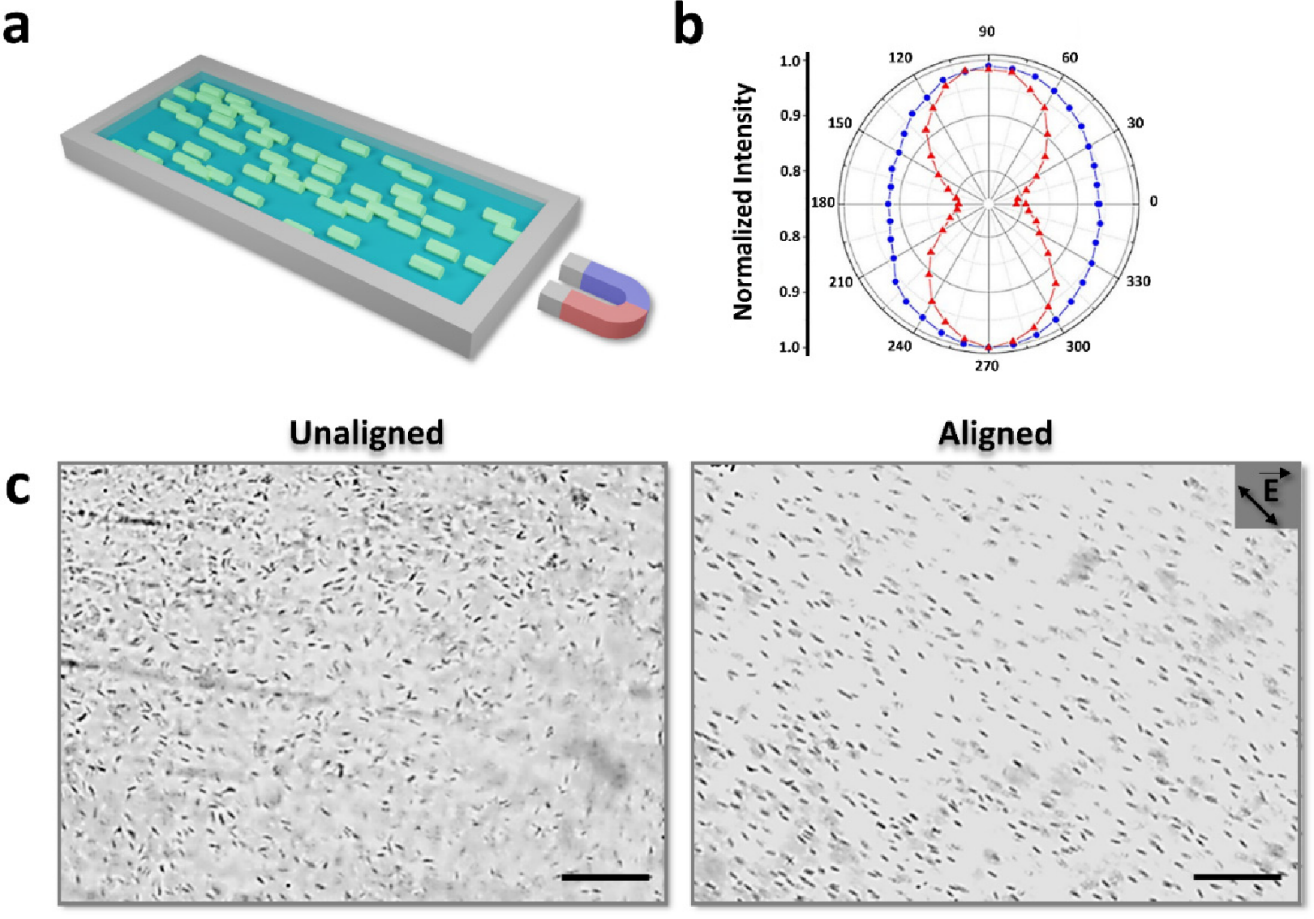
a) Schematic illustration of NU-1000/resin composite preparation. b) Azimuthal plot of fluorescence intensity response of aligned and unaligned NU-1000 crystals in Sylgard 184, to linearly polarized light (blue: unaligned NU-1000, red: aligned NU-1000). c) Optical microscope images of unaligned and aligned NU-1000 crystals in Sylgard 184. Scale bars: 200 μm. Adapted with permission from ref (3). Copyright 2017, John Wiley and Sons.

Optical microscopy observations showed that magnetized NH_2_-MIL-53(Al) crystals could rapidly align when a magnetic field was applied, even when dispersed in viscous photocurable resins to fix them in their oriented state. Similarly, Fe_3_O_4_-coated NU-1000 could also be prepared and oriented in the ⟨001⟩ direction, generating aligned NU-1000/polymer composites showing a strong anisotropic response to linearly polarized light (Figure 6).

Besides crystal orientation, magnetic fields can be utilized to control the positioning of MOFs. Falcaro et al. (2011) prepared MOF-5 crystals embedded with Co nanoparticles. The resulting MOF-5 crystals could be dynamically positioned using an external magnet, and utilized to seed further MOF growth.^40^ In another study, Van Essen et al. (2020) synthesized magnetized m-ZIF-8 with embedded Fe_3_O_4_ nanoparticles for CO_2_ gas filtration.^41^ In their study, m-ZIF-8/Matrimid mixture in DMF was exposed to a magnetic field to generate films with ZIF-8 crystals distributed along the magnetic field lines and the Matrimid membranes with aligned composites showed enhanced CO_2_ diffusion compared to those without alignment.

## 3 Potential Applications

**Figure 7:**
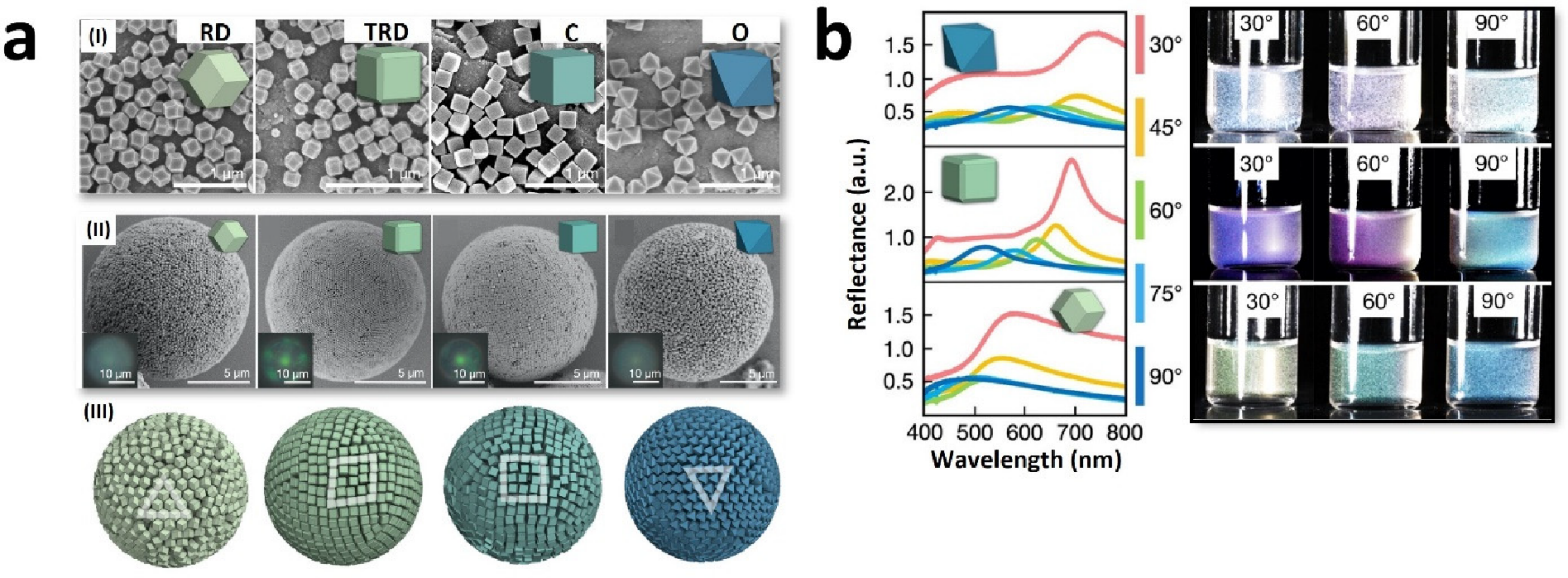
a) FESEM images of rhombic dodecahedron (RD), truncated rhombic dodecahedron (TRD), and cubic (C) ZIF-8 crystals, and octahedron (O) UiO-66 crystals (I), FESEM images of corresponding supraparticles (II), Monte Carlo simulations with polyhedra in spherical confinement (III); b) angle-dependent reflectance spectra of MOF supraparticles with corresponding photographs showing the observable coloration. Adapted with permission from ref (42). Copyright 2022, John Wiley and Sons.

Undoubtedly, the control over MOF crystal morphology and their assembly can open up new avenues for their utilization, such as in photonic applications, whereby the assembly of uniform MOF crystals can generate periodic structures that can interact with light.^26^ Besides flat substrates, liquid droplets can also be utilized to template photonic MOF superstructures. In one of these studies, Wang et al. (2022) functionalized MOFs with different morphologies (truncated- and rhombic dodecahedral ZIF-8, cubic-ZIF-8, and octahedral-UiO-66) using alcohol ethoxylate and PVP, followed by emulsifying these MOFs in perfluorinated oil, thus hierarchically arranging MOF crystals around oil droplets^42^ (Figure 7a). Consequently, these structures served as “*Bragg reflectors*”, inducing interference effects resulting in structural coloration.

**Figure 8:**
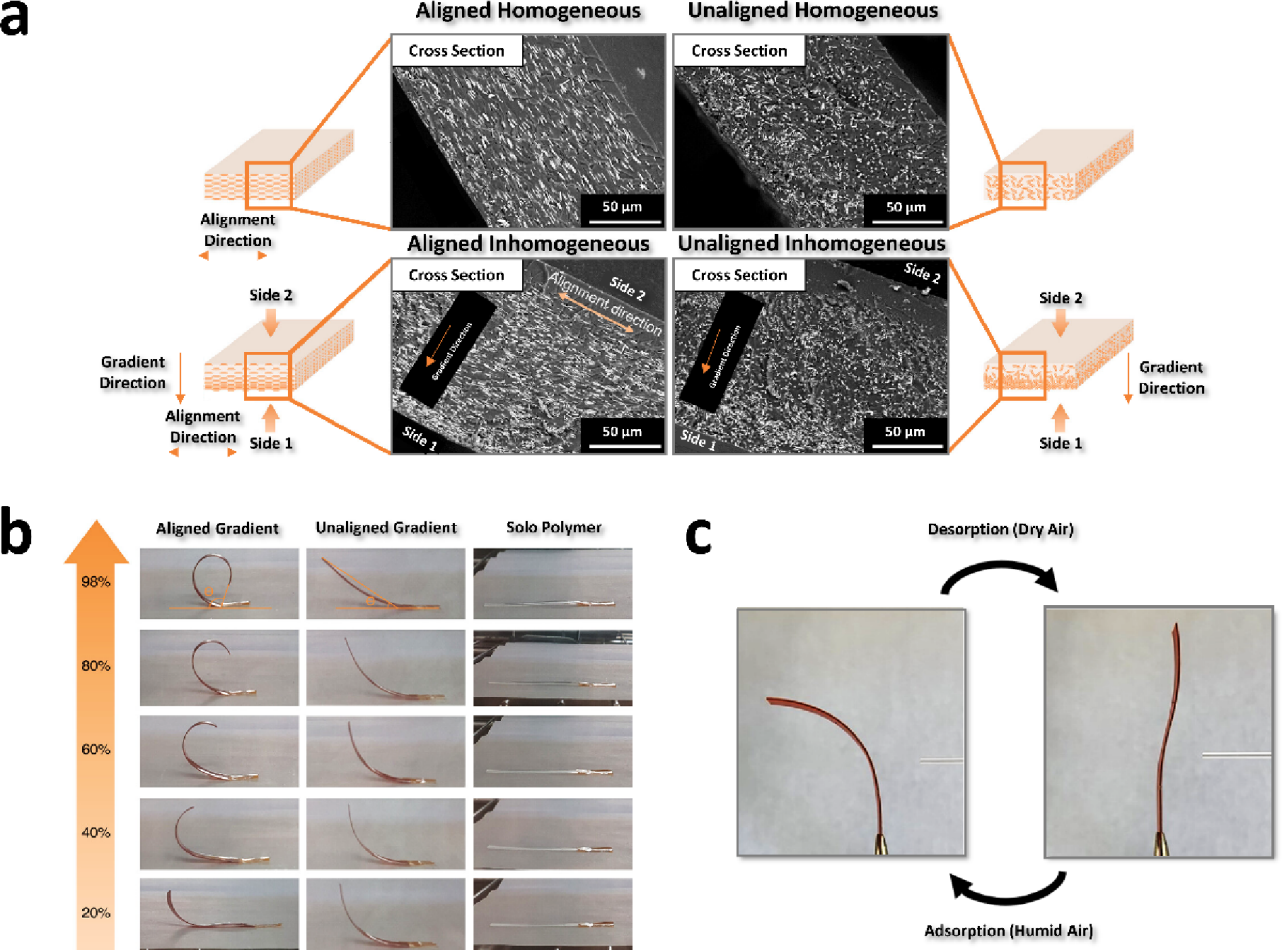
Use of polymer matrices containing aligned MOF crystals as humidity sensors: a) SEM images of aligned and unaligned films of MIL-88A. b) Actuation behavior of the films (aligned-gradient and unaligned-gradient) and polymer at different humidity levels (20–98%). c) Reversible actuation of the aligned-gradient film upon exposure to humid/dry air. Adapted with permission from ref (43). Copyright 2023, American Chemical Society.

The porous structures and photonic properties of MOF superstructures render them particularly valuable for sensing applications since various stimuli such as temperature, pH or guest molecules can trigger expansion or contraction along predetermined crystallographic axes in MOFs. This capability not only qualifies them as sensors but also makes them valuable for use in smart materials such as soft robotics or absorbents. For instance, when exposed to water, MIL-88A exhibits a decrease in the lattice parameter *c* from 15.31 to 12.66 Å and a simultaneous increase in the lattice parameter *a* from 9.26 to 13.87 Å.^43,44^ This property is particularly ideal for the development of moisture-sensitive MOF-based sensors and actuators, as demonstrated by several groups.^45−47^ However, the strong anisotropic lattice changes mean that the expansion and contraction effects of unaligned particles would partially cancel each other out during the directional swelling of films. In the study carried out by our research group (2023), MIL-88A crystals were E-field-aligned within poly(ethylene glycol) diacrylate (PEGDA), and the resulting films exhibited different bending angles at various humidity levels and could revert back to their original state in dry air.^43^ Films with aligned MIL-88A crystals showed faster and more pronounced responses to changes in humidity (Figure 8) during alternating exposure to dry and humid air.

**Figure 9:**
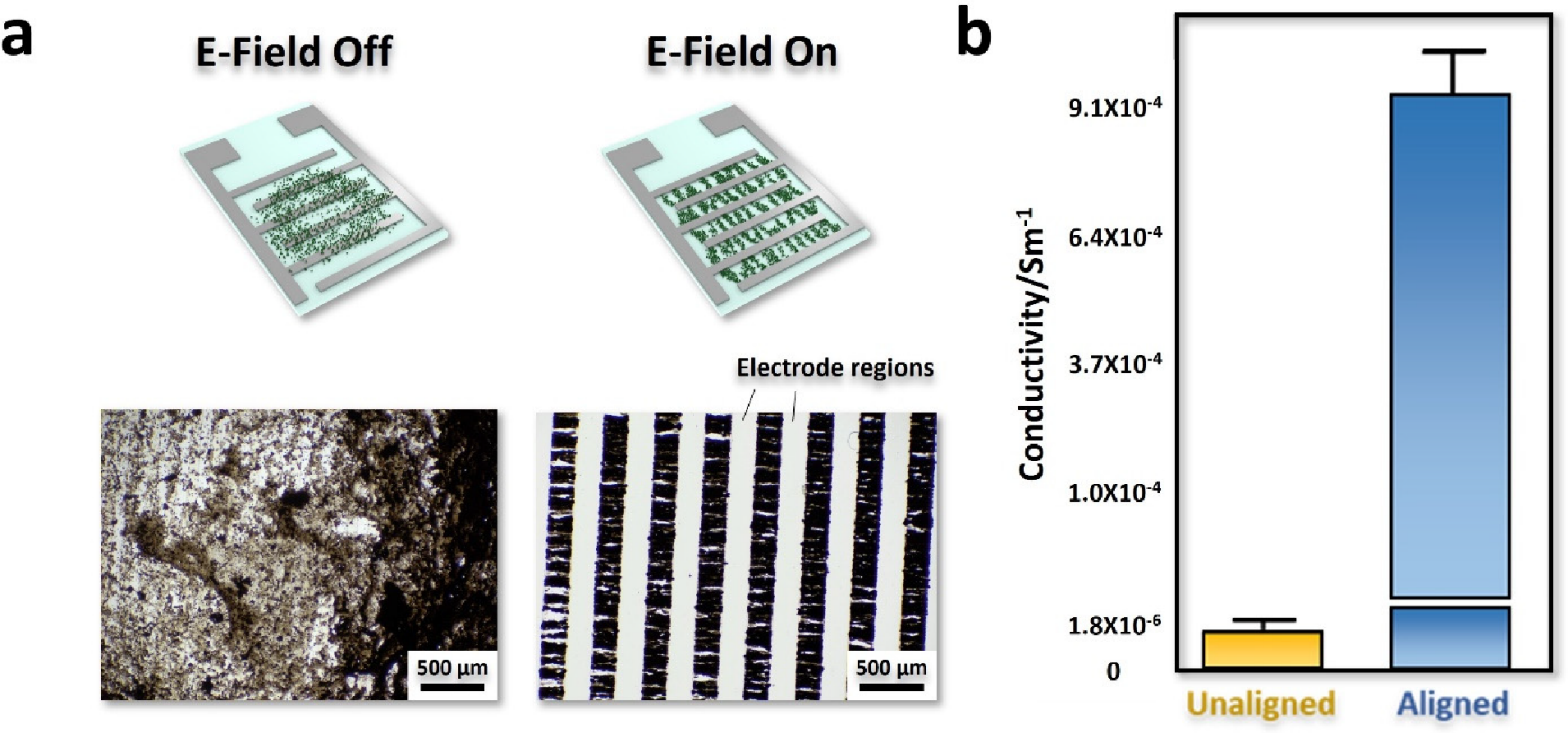
a) Illustration of drop-cast MIL-101(Cr)PEDOT on IDE without and with E-field application (top) and optical microscope images of the corresponding samples showing selective positioning of MIL-101(Cr)_PEDOT_ between the electrodes, appearing as dark stripes when an E-field is applied (bottom). b) Comparison of average conductivity of unaligned and aligned MIL-101(Cr)PEDOT assemblies.

Besides MOF alignment, recently, our group showed that poly(3,4-ethylenedioxythiophene) (PEDOT)-functionalized MIL-101(Cr) can be manipulated by an applied E-field to precisely position the MOF crystals in the interelectrode region on interdigitated electrode substrates during a straightforward drop-casting method, thereby significantly enhancing the degree of control and reliability of the drop-casting process. The MIL-101(Cr)_PEDOT_ crystals rapidly formed chains spanning the interelectrode gaps upon E-field application, in contrast to the randomly deposited crystals when no E-field was applied during drop-casting (Figure 9). The E-field aligned samples showed conductivities more than 500 times higher than the unaligned samples, and enhanced sensitivity for humidity sensing.

Well-organized and aligned MOF crystals also frequently find utility in adsorption and separation applications,^48−51^ as the orientations of MOF crystals can engender significant disparities, particularly in gas separation applications as oriented MOF crystal membranes can exhibit higher gas selectivity compared to membranes with randomly oriented MOF crystals, due to optimized orientation of the gas-sieving pores.

It can be seen that methods to control the crystalline orientation and MOFs, and their spatial positioning whether in films or 3D composites are fundamental to improving their performances in many instances. The exploitation of interfacial confinement and external fields for MOF manipulation offers a practical approach to achieve this.

## 4 Conclusion and Perspective

The increasing recognition that control of MOF morphology, orientation, and assembly is fundamental to the performance of MOFs has led to an array of techniques to structure MOF materials. Effects arising from wettability control determine the positioning of materials at fluid interfaces, which allows localization of MOF crystal growth to achieve unique morphologies or generation of Janus MOF crystals. Assembly of MOF crystal superstructures can be directed via spatial confinement at fluid interfaces, depletion forces, or gravitational sedimentation, allowing a means to achieve the ordering and orientation of MOFs.

Electric field-assisted assembly offers a dynamic approach for rapid and reversible alignment of MOF crystals, with potential applications in electronic devices and reconfigurable systems. Although much insight can be gleaned from the work of others on E-field colloidal manipulation, open questions remain regarding how to enhance MOF polarizability and maximize their E-field response under a range of environmental conditions. While classical colloidal assembly has focused on nonporous colloids such as polystyrene or silica particles, which may have distinct responses from porous MOF crystals, common methods to tune colloidal assembly through the selection of appropriate solvents and the use of different E-field frequencies can be easily adapted for MOFs. However, the molecular designability and porosity unique to MOFs open up new avenues for investigation, for example, through the incorporation of conductive guests to enhance MOF polarizability and E-field response. Through the integration of superparamagnetic particles, magnetic field-assisted assembly can also be applied to a wide variety of MOFs regardless of whether they possess intrinsic magnetic properties, thereby enabling controllable orientation and positioning.

However, it should be noted that in many cases of MOF colloidal assembly, coating of the MOFs with polymer^22^ or surfactants^26,27^ is necessary to avoid particle aggregation, and to enhance the stability of their dispersions as well as to generate ordered assemblies, but presents the potential drawback of MOF pore blockage which can hinder their applicability. To avoid disordered MOF crystal aggregation, future work may benefit from relying upon the use of interparticle electrostatic repulsion rather than pore-blocking steric stabilizers in applications such as sensing or catalysis, whereby MOF porosity retention is crucial. This would allow for the use of less sterically hindered surfactants, or even their avoidance by simply tuning particle zeta potential through the pH of the solvent. Similar considerations apply for the incorporation of additives or guest molecules like Fe_3_O_4_ nanoparticles or PEDOT to enhance MOF responsivity to external magnetic and E-fields, whereby care must be taken that the requisite MOF performance is still present, such as in the case of PEDOT-loaded MIL-101(Cr) for humidity sensing.^52^

The potential applications of these tailored MOF assemblies encompass various fields, including photonics, sensors, gas separation, and smart materials. Particularly, the precise control over MOF crystal orientation and alignment achieved through these assembly methods significantly enhances their performance in the aforementioned applications. Further exploration of these structuring techniques and their applications is warranted to unlock the full potential of MOFs in addressing diverse challenges across multiple disciplines. Therefore, future research efforts of our research group are directed toward deepening our understanding of the external field control of MOFs, refining these assembly methods for improving MOF performances, and exploring novel applications for these exciting materials.
